# Cuteness conquest: how cuteness type and brand impression affect consumers’ brand evaluation in social media

**DOI:** 10.3389/fpsyg.2026.1730776

**Published:** 2026-02-27

**Authors:** Jia Wang, Jia Zhou, Jiawen Shen, Xiaomei Wang

**Affiliations:** School of Journalism and Communication, Hangzhou City University, Hangzhou, China

**Keywords:** brand impression, cuteness, parasocial interaction, social exclusion, social media marketing

## Abstract

While cuteness has become a strategic tool for brands on social media, its effectiveness remains inconsistent. This ambiguity stems from an oversimplified conceptualization of cuteness and an incomplete understanding of the relational mechanisms of its operation. To fill this gap, we developed and tested a model based on consistency theory and stereotype content model (SCM). We distinguish kindchenschema and whimsical cuteness, and examine how their consistency with brand impression (warmth and competence) shapes brand evaluation by promoting parasocial interaction (PSI). The results of three experiments show that a match (e.g., warmth with the kindchenschema, competence with whimsical) promotes PSI, thereby enhancing brand evaluation. Crucially, we identify social exclusion as a key boundary condition: this matching effect is significantly amplified among socially excluded consumers, for whom it serves a compensatory role, but is attenuated among socially included individuals. By moving beyond assessment of the cuteness intensity, this research provides actionable insights for strategically deploying brand personification on social media.

## 1 Introduction

In the landscape of digital marketing, cuteness has evolved from a mere aesthetic flourish into a strategic tool for brand communication (Gretry et al., 2017). Brands increasingly leverage cuteness—manifested through personified mascots, whimsical visual designs, affectionate communication styles, and playful interactions—on social media platforms to capture attention (Kringelbach et al., 2016) and foster consumer connections (Yang, 2023; Chen and Jia, 2023; Suci and Wang, 2023). This widespread phenomenon manifests in diverse forms, making it a timely and important subject for research, especially as brands increasingly seek to build genuine engagement in a crowded, relationship-driven social media landscape.

However, using cuteness is risky, and the results are often mixed. While some cute appeals have helped build brand equity (Li and Eastman, 2024) and smoothed over service failures (Lv et al., 2021; Septianto and Kwon, 2022), others can backfire. Sometimes, it makes the brand look incompetent, which actually hurts its reputation (Stavropoulos and Alba, 2018; Yoon et al., 2022; Messer et al., 2024).

To understand these different influences, we need to appreciate the inner nature of cuteness and the unique relationship background of social media. On social platforms, the use of cuteness is not just about triggering a quick emotional reaction. Instead, it focuses more on building long-term relationships through personal interaction (Yim et al., 2024). In this way, consumers start to feel an emotional bond with the brand persona. This creates a sense of one-sided intimacy, known as parasocial interaction (PSI) (Horton and Wohl, 1956; Shen, 2020). This shift from “viewing” to “interacting” fundamentally alters the mechanism of cuteness. It is in this context of establishing relationships that the childishness and dependence schema inherent in cuteness (Lorenz, 1943; Sherman and Haidt, 2011) may conflict with the professional and trustworthy image that the brand wants to convey (Waytz et al., 2010).

Although cute marketing is often studied through the lens of brand personification and schema consistency theory (Aggarwal and McGill, 2007; Jörling et al., 2020; Jeong and Kim, 2021), the existing research mainly focuses on the single prototype of Kindchenschema. This narrow focus fails to explain why many competence-oriented brands (e.g., in technology or finance) successfully leverage whimsical cuteness, which is characterized by humor and quirkiness. The difference between Kindchenschema and whimsical cuteness (Nenkov and Scott, 2014) has been acknowledged, and its differential effect in the presentation environment has been verified (Ye and Shi, 2020; Feng et al., 2022); however, this research idea has not been systematically applied to social media.

Furthermore, distinguishing between cuteness types makes their congruence with brand traits a critical factor. However, most studies on the matching effect conceptualize it as a static cognitive mechanism that directly affects aesthetics or product evaluation, mainly focusing on product attributes or specific behavior areas [for example, tourism destination evaluation, Li and Zhou (2023); Pro-environmental behavior, Xu et al. (2025)]. This static perspective overlooks the essence of social media marketing. Social platforms are defined by dynamic, two-way interactions focused on relationships. On these platforms, consumers do not merely evaluate a product; they interact with the brand almost like a social partner. Consequently, the way cuteness functions differs and becomes more evident in social media contexts. Marketing effectiveness depends less on direct persuasion and more on cultivating such connections. Simply applying insights from earlier studies on packaging or product designing is insufficient, as this overlooks the relationship-oriented nature of social platforms. Thus, we must reconsider the role of consistency: only when the style of cuteness authentically aligns with the brand’s identity will consumers transition from passive viewers to active participants in PSI.

To address these gaps, we propose a relationship-centered mediation framework that moves beyond a static understanding of matching effects. Using the stereotype content model (SCM), we operationalize brand impression into two main dimensions: warmth and competence (Fiske and Neuberg, 1990) and examine how they relate to kindchenschema and whimsical cuteness. This approach shifts the matching logic from simple ‘attribute consistency’ to a more human-like ‘personified impression consistency,’ while pointing out the key role of PSI. This fits the relationship-focused nature of social media well and broadens the understanding of matching effects in this field.

Besides brand strategy, the effect of being cute also depends on the consumer’s state of mind (Nittono and Ihara, 2017). Belonging is a basic need, and feeling socially excluded can deeply affect how people think and behave (Gardner et al., 2000; Wang et al., 2017). People who feel excluded are more sensitive to signs of connection (Maner et al., 2007), so they might react differently to different types of cuteness. Previous studies on relationships (Gardner et al., 2000), brand personification (Chen and Yang, 2017), and tourism (Su and Li, 2023) have all noted this shift. Since kindchenschema is biologically known to trigger attachment (Lorenz, 1943; Yim et al., 2024), we assume socially excluded consumers might find more comfort in it. Therefore, it is important to look at the consumer’s social context as a moderating factor.

In summary, as brands increasingly invest in building relationships on social media and as cute appeals become more common yet risky, this study responds to the absence of a clear system for linking types of cuteness with varied brand impressions on these platforms. By clarifying how different forms of cuteness relate to brand perceptions in a relationship-based context, the research provides practical and timely insights for marketers who wish to use cuteness strategically while reducing its potential drawbacks.

To provide these insights, this study aims to: (1) shift the focus from product to brand impression matching; (2) reconceptualize the core mechanism from improving evaluation to establishing relationships, and explain how matching indirectly affects brand evaluation through PSI; and (3) contextualize the matching effect by examining social exclusion as a moderator, revealing how the efficacy of cuteness marketing depends on consumers’ psychological needs.

To this end, we designed three experiments: Study 1 examined the baseline effect of different cute appeals (kindness, whimsical and a non cute control) on PSI; In Study 2, brand impression (warmth and competence) was introduced to investigate the intermediary path of matching → PSI → brand evaluation. Study 3 further incorporates a social exclusion scenario to explore the boundary conditions of this pathway. Through these studies, we aim to integrate the consistency theory and SCM into the context of social media anthropomorphism, so as to build a more refined and dynamic theoretical framework to understand cute marketing.

## 2 Literature review

### 2.1 Cuteness appeals and PSI

In social media, the relationship between brands and consumers is increasingly established through emotional and anthropomorphic communication (Labrecque, 2014). This method uses personification, that is, humans tend to attribute intentions and emotions to non-human entities such as brands (Epley et al., 2007). Parasocial interaction (PSI), a concept from media studies, describes the one-sided, personal connection an audience feels toward media figures (Horton and Wohl, 1956). This concept is also applicable to social media, in which brand accounts usually play a media role with unique and continuous personality (Labrecque, 2014). The concept of computer as a social actor (CASA) holds that people unconsciously use social norms to interact with these brands (Nass and Moon, 2000). Therefore, consumers may evaluate the brand’s personality and behavior as they do their human peers. This perceived one-sided social relationship is the essence of PSI (Horton and Wohl, 1956).

#### 2.1.1 The dual nature of cuteness

Although cute appeal can effectively trigger personification and PSI, cuteness itself is not a single structure. Existing research indicates that cuteness stems from two distinct types that diverge significantly in their evolutionary origins, neural mechanisms, and cognitive processing pathways: kindchenschema cuteness and whimsical cuteness.

Specifically, kindchenschema cuteness is rooted in the evolutionary “Baby Schema” (Lorenz, 1943), characterized by a specific set of infantile morphological features such as round faces, large eyes, and small noses. Neuroimaging studies demonstrate that viewing such faces rapidly activates brain regions associated with reward, emotion, and attention (e.g., the nucleus accumbens and orbitofrontal cortex), eliciting feelings of warmth and triggering caretaking and approach impulses. Functioning as an Innate Releasing Mechanism (Kringelbach et al., 2016), this response precedes complex cognitive evaluation and directly activates the nurturant instinct and desire to protect. Consequently, its core social function lies in signaling vulnerability and harmlessness (Sherman and Haidt, 2011).

In contrast, whimsical cuteness is more closely aligned with the evolution of play, humor, and human intelligence. Rather than relying on fixed morphological features, it elicits liking through cognitive incongruity, wit, or unexpected playfulness (Nenkov and Scott, 2014; Gorn et al., 2008). Its cognitive processing involves evaluation and comprehension recruited by the prefrontal cortex, requiring the resolution of unconventional elements and the appreciation of humor (Warren and McGraw, 2016). This pleasure comes from cognition, not biological instinct. Therefore, it reflects cognitive flexibility and play interaction rather than simple vulnerability (Li et al., 2022).

#### 2.1.2 Cuteness appeals as antecedents of PSI

The link between cuteness and PSI rests on two connected theories. First, the theory of anthropomorphism posits that people tend to attribute human characteristics to nonhuman objects to facilitate easier connection with them (Epley et al., 2007).

Cuteness, whether kindchenschema or whimsical, makes brands appear warm, vulnerable, or playful—all traits that resonate with human patterns. Second, the CASA perspective suggests that people unconsciously perceive mediated entities as social beings and apply social norms to them (Nass and Moon, 2000). Cute brand cues activate social scripts such as caring or companionship, prompting consumers to engage in social interactions rather than transactions. In essence, personification induced by cuteness and social engagement forms the foundation of PSI. Compared with a plain informational appeal, a cute one lowers the barrier to social perception and invites relational interaction. Therefore, we hypothesize:

*H1a:* Brand appeals featuring cuteness will elicit higher levels of PSI than non-cute appeals.

In addition, cuteness is a matter of degree, not an all or nothing quality. Emotionalism shows that more stimuli will trigger more emotional responses (Lazarus, 1991). Applying this principle, a brand perceived as cuter should evoke positive emotions and a more intense approach motivation. This strengthened emotional connection should also foster a higher level of PSI. Therefore, we assume that:

*H1b:* The cuteness degree of the brand is positively correlated with the PSI level.

### 2.2 The matching effect of cuteness types and brand impressions

The effectiveness of cute appeal on social media is significantly affected by consumers’ pre-existing brand impressions (Li and Eastman, 2024). Although the Consistency Theory holds that individuals tend to accept information consistent with their existing cognitive schema (Osgood and Tannenbaum, 1955), it only regards cute marketing as a simple visual match, ignoring the uniqueness of cute as a special psychological stimulus.

Unlike neutral product shapes or functional attributes, cuteness is a potent relational social signal (Shen, 2020). Its core function is to activate specific social relational scripts and role expectations. Therefore, when a brand launches a cute appeal, it initiates a relationship invitation, inviting consumers to enter a defined emotional role play (e.g., caregiver or playful companion). Whether consumers will accept the invitation depends on the overall impression of the brand they have formed (Gidaković et al., 2021). According to SCM, brand impressions is mainly composed of two basic dimensions: “warmth” and “competence” (Fiske and Neuberg, 1990): the former conveys sincerity and friendliness, while the latter reflects efficiency and skills. It is these two dimensions that constitute the core of understanding of personified brands (Kervyn et al., 2012).

SCM provides a key perspective for us to understand the differentiation effect of two cute types (Fiske and Neuberg, 1990). Kindchenschema cuteness is rooted in theevolutionary formed”baby schema” (Lorenz, 1943), which can automatically transmit signals that are harmless, fragile and need to be cared for (Sherman and Haidt, 2011). Therefore, this signal can naturally fit with the role of “caregiver” that warm brands try to shape, so as to effectively strengthen its sense of authenticity as a trusted partner. In contrast, the core mechanism of whimsical cuteness lies in resolving cognitive dissonance through humor and wit (Nenkov and Scott, 2014), conveying signals of fun, creativity and a sense of intellectual participation. This is more naturally in line with the role of competence brand that aim to demonstrate efficiency and wisdom. It can not only show a surplus of capabilities (Puzakova and Aggarwal, 2018), but also avoid the risk of appearing naive (Shin and Mattila, 2021).

Empirical research has repeatedly confirmed the effect distinction between these two types of cute: whimsical cuteness usually performs better in improving perception (Ye and Shi, 2020), improving product quality perception (Li et al., 2022) or in luxury marketing and other scenarios that emphasize ability (Feng et al., 2022); On the contrary, in the scene where trust and emotional connection need to be established, the kindchenschema cuteness has more advantages (Sun et al., 2025). Notably, a recent study affirms that the effect of kindchenschema (vs. whimsical cuteness) is specific to shaping particular sensory expectations, such as sweetness, thereby driving consumer choice (Bruckdorfer and Büttner, 2025). The reason for this difference lies in the fact that the two are, respectively, rooted in emotional instinct and cognitive assessment, which determines that they will be combined with different dimensions of brand impression in a targeted manner.

In summary, this study proposes the “Cuteness-Impression Consistency Model, “whose core is to deepen the matching logic from the traditional level of functional attributes to the fit between relationship signals and social roles. The model advocates that the maximization of brand evaluation depends on the coherence between the interactive script implied by the cuteness type and the social role carried by the brand impression. From this, we derive the following assumption:

*H2a:* For a brand perceived as warm, using kindchenschema (vs. whimsical) cuteness will lead to more positive brand evaluation.

*H2b:* For a brand perceived as competent, using whimsical (vs. kindchenschema) cuteness will lead to more positive brand evaluation.

### 2.3 The mediating role of PSI

As mentioned above, the matching between cuteness type and brand impression constitutes the consistency between relationship signals and social roles. This deep-seated consistency not only defines the internal credibility of the personalised role of the brand, but also triggers a series of predictable and positive psychological reactions in the process of consumer information processing. These psychological reactions form the bridge through which the matching effect is transformed into a sustainable, mediated interpersonal relationship—namely, PSI.

Specifically, when the cuteness type is successfully aligned with the expected brand role (for example, the whimsical cuteness and “capable partner”), this relationship consistency first produces a smooth cognitive experience: information becomes easier to process and classify. According to processing fluency theory (Reber et al., 2004), the experience of fluency itself can automatically elicit positive affect (Winkielman and Cacioppo, 2001). Further research indicates that the positive affect arising from fluent processing of a stimulus is directly translated into preferences and favorable attractiveness evaluations of that stimulus (Winkielman et al., 2006). In the context of anthropomorphism, consumers tend to mistakenly attribute the pleasure brought by smooth processing to the social attraction of brand personas (Halberstadt, 2006). Therefore, processing fluency is not a simple explanation for the matching effect; rather, it is the concrete psycho-affective mechanism through which successful relational congruence operates at the individual level. It transforms abstract role fit into tangible attraction and authenticity, thereby providing the direct psychological impetus for PSI to develop.

In addition, the attractiveness and logical coherence generated by successful matching jointly construct the anthropomorphic authenticity of perception. This sense of authenticity enhances perception of credibility and predictability, thus effectively reducing consumers’ psychological defense in social media interaction (Beverland and Farrelly, 2010). When users no longer question the plausibility of the brand persona, they become more willing to suspend disbelief and proactively engage with the brand’s interactive invitations (Escalas and Bettman, 2017). This shift from appreciation to participation in interaction encourages users to invest emotional resources in behavior, such as comments and likes (Golonka et al., 2023), thereby fostering a one-sided intimate bond—the core of PSI formation. Based on this reasoning, we propose the following hypothesis:

*H3:* PSI mediates the interactive effect of cuteness type and brand impression on brand evaluation.

### 2.4 The moderating role of social exclusion

Social exclusion is a strong negative experience that is ignored or rejected, which directly threatens the basic belonging needs of human beings (Baumeister and Leary, 1995; Williams, 2007). Acting as a compensatory mechanism, this experience sensitizes individuals to cues of social warmth (Maner et al., 2007). Specifically, excluded individuals prioritize social information (Gardner et al., 2000), cultivate deeper PSI with media figures (Dibble et al., 2016), and demonstrate heightened attachment needs (Schoch et al., 2015). This drive extends to consumption, where individuals seek emotional reconnection through nostalgic products (Wu and Jiang, 2019) or warm, personified brands (Chen and Yang, 2017).

However, the precise role of social exclusion in the context of cute marketing remains largely unexplored. Although some studies have provided preliminary clues-for example, cuteness can have a greater “healing effect” on excluded individuals (Su and Li, 2023), or that consumers with strong motivational tendencies are more sensitive to kindchenschema clues (Wang et al., 2017) - but lack of systematic understanding. Specifically, it is unclear how social exclusion might alter consumer responses to different types of cuteness, especially when considering the congruity with a brand’s established impression. This study aims to address this theoretical gap.

According to the Compensatory Consumption Theory, we believe that consumers’ choices are usually driven by the need to repair psychological deficits (Sivanathan and Pettit, 2010). For an individual experiencing a deficit in belonging, the warmth and caregiving instincts evoked by kindchenschema cuteness represent the most direct and potent signal of social connection available. Therefore, we predict that social exclusion will amplify the need for such warmth cues. Consequently, the positive effect of the “warm-brand and kindchenschema-cuteness” match will differ significantly between groups, being more favorable among excluded individuals. This specific combination offers the most effective way to restore their damaged sense of belonging and alleviate negative feelings (Chou et al., 2022; Lv et al., 2021). For socially included consumers, in contrast, the same content might simply be perceived as amusing rather than emotionally restorative. We therefore propose:

*H4:* Social exclusion moderates the interactive effect of cuteness type and brand impression. Specifically, for a brand perceived as warm, the positive effect of kindchenschema (vs. whimsical) cuteness on brand evaluation will be more significant for socially excluded consumers than for socially included consumers.

The dynamic may differ for a brand known for its competence. When an excluded individual encounters content from such a brand, their psychological response is less certain. If the competent brand uses kindchenschema cuteness, the warmth signal might feel forced or inauthentic due to the incongruity with its core image, potentially failing to provide comfort. In contrast, whimsical cuteness repairs mood through humor. This approach relies on distraction and amusement, rather than fulfilling a need for warmth. The direction and strength of this effect are theoretically ambiguous. Therefore, we pose this as an exploratory research question:

*RQ:* For a brand perceived as competent, does social exclusion influence how consumers evaluate kindchenschema cuteness versus whimsical cuteness?

**Figure 1:**
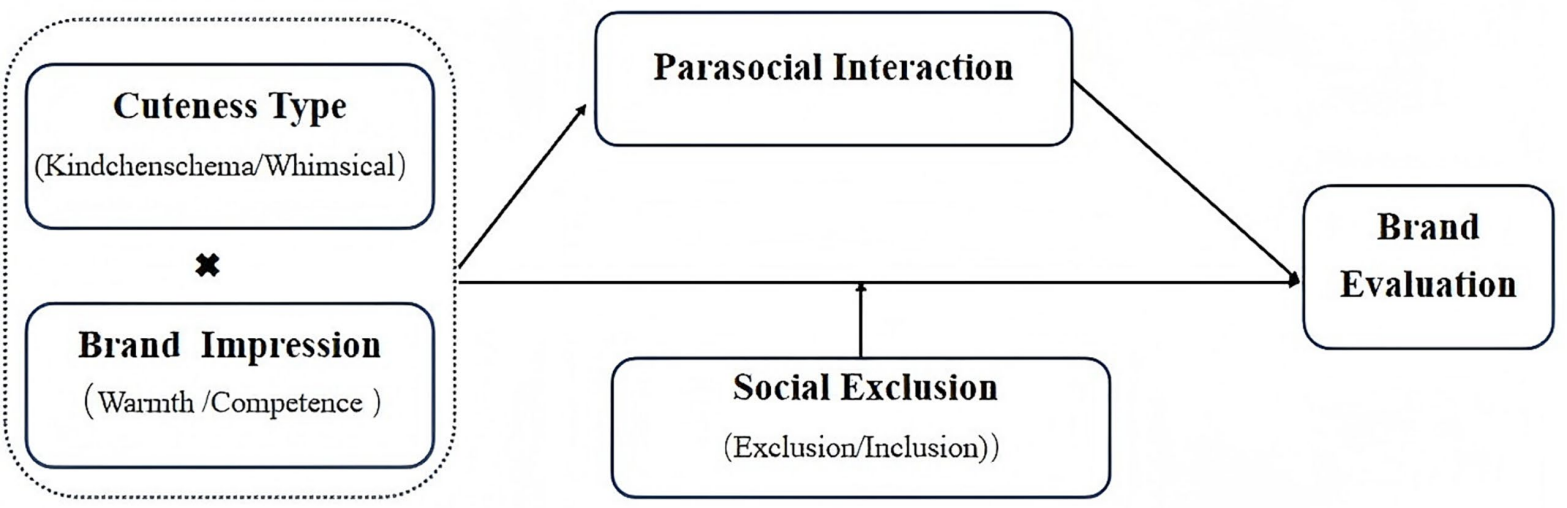
Conceptual model.

Figure 1 displays the study’s conceptual model.

## 3 Study 1: the main effect of cuteness appeals

We conducted a one-factor experiment to assess the impact of different cute stimuli (kindchenschema, whimsical, vs. a non-cute control) on PSI. The study aimed to determine if cute appeals elicit greater PSI than non-cute ones (H1a), verify a positive relationship between perceived cuteness and PSI (H1b), and explore any significant difference in PSI between the two cuteness types.

### 3.1 Design and participants

The questionnaire survey was conducted via an online platform^1^. Data screening rules were established to assure high-quality collecting. After excluding subjects who did not pass the attention check and logical verification tests, 240 valid responses were obtained from participants (M_age_ = 30.14 years, *SD* = 8.12 years; 101 men, 139 women) and placed in one of the above three conditions. All studies were conducted within a simulated scenario of the Chinese social media platform Weibo. The platform type was held constant across all experiments and was not manipulated as a variable.

### 3.2 Procedures and manipulations

Participants first reported their baseline emotional state [i.e., positive and negative affect using items from Watson et al. (1988)] before being assigned to one of three experimental conditions randomly. Subsequently, they were instructed to imagine themselves considering purchasing pour-over coffee and came across the Weibo page of a fictional brand “Niso Coffee.” Coffee was selected as the product category due to its affectively neutral and low-involvement nature (e.g., Dhar and Wertenbroch, 2000; Chao, 2022), which helps to isolate the effects of the cute appeals.

The experimental stimuli consisted of three versions of the Weibo homepage. Following the approach of Yoon et al. (2022), all non-critical elements (e.g., links, videos, interaction data) were removed to focus solely on the core appeals. The stimuli were desgined to capture the psychological essence of each cuteness type and to ensure each dimension was distinctly and fully represented. The kindchenschema cuteness stimulus aims to activate nurturing instincts. It featured visuals, emojis, and text aligned with infant schemas (e.g., round eyes). The whimsical cuteness stimulus aims to evoke funny and humor. It utilized funny emojis, witty text, and unconventional design elements (e.g., “eye masks”) to convey a quirky and playful tone. The control (no cute elements) condition employed a neutral, product-focused style with standard product photos and objective descriptions. Full stimulus materials are presented in Supplementary Appendix A. After viewing their assigned page for at least 30 s, participants completed a questionnaire measuring the dependent variables, manipulation checks, and demographic information.

### 3.3 Variable measurement

A 7-point Likert scale (1 = strongly disagree, 7 = strongly agree) was employed across all studies. Perceived cuteness is a holistic, evaluative construct that reflects an individual’s overall judgment of how “cute” a stimulus is, reflecting the intensity or degree of cuteness. It was measured using three items adapted from Nenkov and Scott (2014): cute, adorable, endearing (*α* = 0.84). Kindchenschema schema cuteness and whimsical cuteness are analytical, descriptive constructs that refer to the specific style or type in which cuteness is expressed—that is, the manner of cuteness. Also drawing on Nenkov and Scott's (2014), kindchenschema cuteness was measured through “vulnerable, naive, and caretaking” (*α* = 0.80), whereas whimsical cuteness was measured with “whimsical, playful, and fun” (*α* = 0.81). Although some terms may appear to overlap with general cuteness judgments, when presented within distinct theoretical constructs and scale sets, participants distinguish them based on specific instructions. This approach has been applied in previous research (Nenkov and Scott, 2014; Ye and Shi, 2020; Feng et al., 2022; Yim et al., 2024; Sun et al., 2025). Emotional state was measured with four items from Watson et al. (1988) (*α* = 0.84). To measure PSI, we used a scale from Shen (2020), which was adapted from foundational work by Perse and Rubin (1989) and Rubin et al. (1985) (*α* = 0.83). Finally, participants provided their demographic information. All scale items are listed in Supplementary Appendix B.

### 3.4 Results

#### 3.4.1 Discriminant validity of the measurement scales

First, to establish the discriminant validity of our core constructs—perceived cuteness, kindchenschema cuteness, and whimsical cuteness—we conducted a confirmatory factor analysis (CFA). We compared three competing models: a three-factor baseline model, a two-factor model (combining kindchenschema and whimsical cuteness), and a one-factor model. As shown in Table 1, the three-factor model demonstrated good fit to the data (χ^2^/df < 2, CFI > 0.99, RMSEA ≈ 0). In contrast, both the two-factor and one-factor models showed substantially poorer fit. Chi-square difference tests confirmed that the three-factor model fit significantly better than the two-factor model [Δχ^2^(2) = 60.114, *p* < 0.001] and the one-factor model [Δχ^2^(3) = 89.609, *p* < 0.001]. Furthermore, The square root of the AVE for each construct (ranging from 0.71 to 0.82) exceeded its correlations with the other constructs. These results confirm that the three constructs are psychometrically distinct.

**Table 1:** Comparison of fit indices for competing CFA Models in Study 1.

Model	χ^2^/df	CFI	TLI	RMSEA	SRMR
Three-factor model	0.98	1.00	1.00	0.00	0.10
Two-factor model	3.21	0.77	0.69	0.15	0.20
One-factor model	4.19	0.66	0.55	0.18	0.17

#### 3.4.2 Experimental manipulations

As intended, participants in the kindchenschema condition reported higher perceptions of kindchenschema cuteness (*M* = 4.94, *SD* = 0.54) than those in the whimsical condition (*M* = 4.35, *SD* = 1.29), *t* (158) = 3.75, *p* < 0.001. Conversely, the whimsical group rated their stimuli as more whimsically cute (*M* = 5.43, *SD* = 0.87) than the kindchenschema group did (*M* = 3.61, *SD* = 0.65), *t* (158) = −15.03, *p* < 0.001.

A one-way ANOVA on perceived cuteness revealed a significant effect of the experimental condition, *F* (2, 237) = 43.52, *p* < 0.001. Post-hoc tests confirmed that participants in the kindchenschema (*M* = 5.78) and whimsical cuteness (*M* = 6.03) conditions perceived the stimuli as significantly more cuteness than those in the control condition (*M* = 4.58). This indicates our cuteness manipulation was successful.

Finally, the groups did not differ on initial mood, *F* (2, 237) = 0.10, *p* = 0.904, or brand familiarity, *F* (2, 237) = 0.28, *p* = 0.758, ruling them out as potential confounds.

#### 3.4.3 Gender distribution and control

A total of 240 participants (101 men, 139 women) were included in the Study 1. Independent-samples t-tests showed no significant gender differences in PSI (*p* = 0.372) or perceived cuteness (*p* = 0.952). To ensure the robustness of the results, gender was controlled for as a covariate in the main analyses. A one-way ANCOVA performed on PSI confirmed that the main effect of condition remained significant, *F*(2, 236) = 5.50, *p* = 0.005, while gender was not a significant covariate (*p* = 0.305). Similarly, a hierarchical regression predicting PSI showed that after controlling for the non-significant effect of gender (*p* = 0.338), perceived cuteness remained a strong positive predictor (*β* = 0.43, *p* < 0.001). These results confirm that gender did not significantly influence the findings related to H1a and H1b.

#### 3.4.4 Hypothesis testing

To test H1a, a one-way ANOVA was performed on PSI. The results showed the main effect is significant, *F*(2,237) = 5.39, *p* = 0.005. Specifically, participants exposed to the kindchenschema (*M* = 4.99, *SD* = 1.09) and whimsical (*M* = 5.08, *SD* = 0.55) stimuli reported significantly higher PSI than those in the control group (*M* = 4.60, *SD* = 1.15). These results support H1a.

To test H1b, a linear regression analysis was performed. The results showed that perceived cuteness significantly and positively predicted PSI (*β* = 0.43, *t* = 7.35, *p* < 0.001), supporting H1b.

## 4 Study 2: The matching effect of cuteness type and brand impression

Study 2 employed a 2 (brand impression: warmth vs. competence) × 2 (cuteness type: kindchenschema vs. whimsical) between-subjects design. Its primary goal was to test the interaction between brand impression and cuteness type on brand evaluation (H2a and H2b). Additionally, we examined the underlying mechanism by testing the mediating role of PSI (H3).

### 4.1 Design and participants

We recruited 321 participants (M_age_ = 29.58, *SD* = 6.42; 110 male) from a major Chinese online survey platform (WJX.cn). The final sample consisted of participants after excluding those who failed embedded attention checks. Participants were randomly assigned to one of the four experimental conditions.

### 4.2 Procedure and manipulations

The procedure was similar to Study 1. After reporting their baseline emotional state, participants were randomly assigned to one of the four conditions. We created four versions of a fictional brand’s Weibo page to manipulate brand impression and cuteness type.

#### 4.2.1 Brand impression

We manipulated brand impression by varying the product category and brand description. The warmth brand, “Mizhiyun,” was a honey company. Its profile emphasized “pure and natural” to convey a gentle and sincere image. The competence brand, “Zhihuijie,” was a smartphone company. Its profile highlighted “cutting-edge technology and exceptional performance” to convey an image of innovation and industry leadership. This design choice was based on the Continuum Model of Impression Formation (Fiske and Neuberg, 1990). According to this model, people form impressions of a brand in two ways: by quickly categorizing it and by paying attention to its specific features. Typically, consumers first use product categories as a mental framework to understand a brand’s attributes, which helps them form an overall impression. The approach of using distinctly different stimuli to represent a concept is well established in prior consumer behavior studies (e.g., Park et al., 1986; Chandon et al., 2000; Bellezza et al., 2014). Crucially, to ensure that the observed effects are attributable to the psychological brand impression rather than the product category *per se*, we (a) held all other stimulus elements (platform, layout, cuteness type) constant, and (b) included direct manipulation checks for perceived warmth and competence to validate the success of our manipulation on the intended constructs.

#### 4.2.2 Cuteness type

The manipulation of cuteness type (kindchenschema vs. whimsical) was identical to that in Study 1.

After viewing the assigned page for at least 30 s, participants completed a questionnaire containing dependent variable measurement, manipulation test and demographic information.

### 4.3 Variable measurement

The measurement item of brand impression is adapted from the scale developed by kervyn et al. (2012). Two items measured brand warmth (e.g., “warm,” “friendly”; *α* = 0.83) and two measured brand competence (e.g., “competent,” “capable”; *α* = 0.91). Brand evaluation was assessed using Aaker’s (1997) five-item scale (*α* = 0 0.87). Measures for kindchenschema cuteness (*α* = 0.87), whimsical cuteness (*α* = 0.89), perceived cuteness (*α* = 0.81), and PSI (*α* = 0.80) were identical to those in Study 1. We also included emotional state (*α* = 0.89) and brand familiarity as control variables, using the same measures from Study 1. Finally, participants provided demographic information.

### 4.4 Results

#### 4.4.1 Discriminant validity of the measurement scales

The three-factor model again showed acceptable fit (χ^2^/df = 1.76, CFI = 0.97, RMSEA = 0.09) and fitted the data significantly better than both the two-factor model [Δχ^2^(2) = 59.04, *p* < 0.001] and the one-factor model [Δχ^2^(3) = 156.48, *p* < 0.001]. The square root of the AVE for each construct (ranging from 0.76 to 0.91) exceeded its correlations with the other constructs, supporting discriminant validity.

#### 4.4.2 Manipulation and confound checks

The manipulations were successful. For cuteness type, a t-test confirmed that participants in the kindchenschema condition reported higher kindchenschema cuteness perceptions (*M* = 4.64, *SD* = 0.93) than those in the whimsical condition (*M* = 3.90, *SD* = 1.05), *t* (319) = 6.78, *p* < 0.001. Conversely, the whimsical group rated their stimuli as more whimsically cute (*M* = 4.32, *SD* = 0.94) than the kindchenschema group did (*M* = 3.45, *SD* = 1.11), *t* (314.37) = −7.56, *p* < 0.001.

For brand impression, the warmth brand was rated as significantly warmer (*M* = 4.88, *SD* = 0.85) than the competence brand (*M* = 3.81, *SD* = 1.17), *t* (301.13) = 9.42, *p* < 0.001. Conversely, the competence brand was rated as more competent (*M* = 5.06, *SD* = 0.97) than the warmth brand (*M* = 3.84, *SD* = 1.28), *t*(285.82) = −9.59, *p* < 0.001.

Importantly, the four experimental groups did not differ in initial emotional state, *F* (3, 317) = 1.72, *p* = 0.163, or brand familiarity, *F* (3, 317) = 1.68, *p* = 0.172, ruling these out as potential confounds.

#### 4.4.3 Hypothesis testing

First, replicating the findings from Study 1, a linear regression showed that perceived cuteness positively predicted PSI (*β* = 0.21, *t* = 3.83, *p* < 0.001), further supporting H1b.

**Figure 2:**
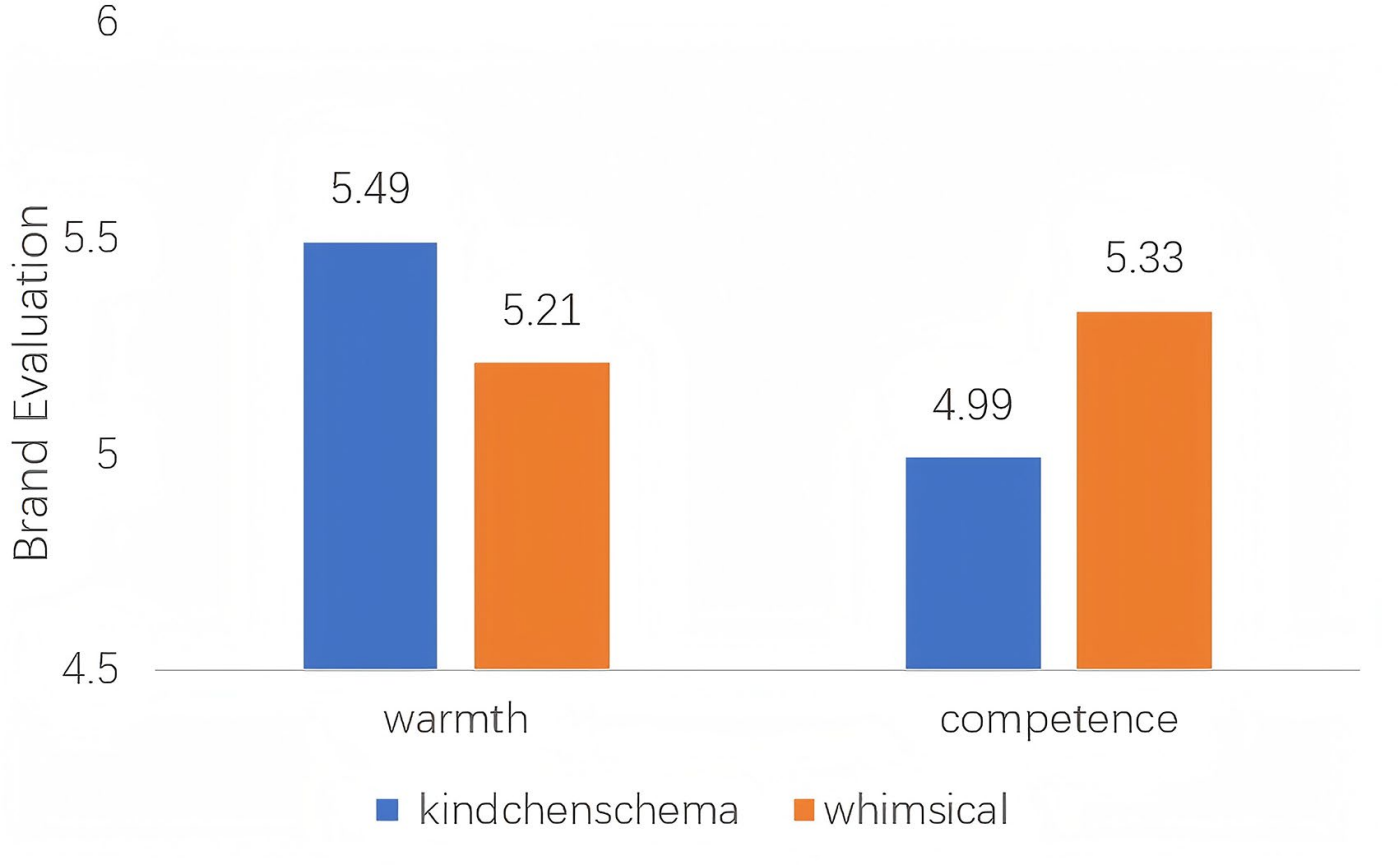
Interaction effect of cuteness type and brand impression on brand evaluation.

To test our main hypotheses, we conducted a 2 (cuteness type) × 2 (brand impression) ANOVA on brand evaluation. The analysis revealed no significant main effect for either cuteness type (*p* = 0.799) or brand impression (*p* = 0.069). However, as predicted, there was a significant interaction effect, *F* (1,317) = 9.65, *p* = 0.002, *η*^2^ = 0.03. We decomposed this interaction using simple effects analysis (see Figure 2). For the warmth brand, kindchenschema cuteness (*M* = 5.49, *SD* = 0.84) led to significantly higher brand evaluation than whimsical cuteness (*M* = 5.21, *SD* = 0.89), *F* (1, 317) = 3.92, *p* = 0.048, *η^2^* = 0.01. This supports H2a. For the competence brand, whimsical cuteness (*M* = 5.33, *SD* = 0.90) led to significantly higher brand evaluation than kindchenschema cuteness (*M* = 4.99, *SD* = 0.94), *F* (1, 317) = 4.68, *p* = 0 0.016, *η^2^* = 0.02. This supports H2b.

To rule out the possibility that the observed effect was driven merely by differences in the overall perceived cuteness level between the two cute conditions (rather than their distinct types), we conducted a robustness check. We performed an analysis of covariance (ANCOVA) using perceived cuteness as a covariate. The results indicated a significant effect for the covariate, *F*(1, 317) = 14.25, *p* < 0.001, confirming that perceived cuteness itself is a significant predictor of brand evaluation. Importantly, after controlling the overall level of perceived cuteness, the interaction effect of “cuteness type × brand impression” was still significant, *F*(1, 317) = 11.47, *p* = 0.001. This shows that a particular type of cuteness is not only “whether something is cute” or “how cute it is”, but also has a unique explanatory power in the interaction with the brand impressions.

#### 4.4.4 Moderated mediation analysis

To test for moderated mediation (H3), we used the PROCESS macro of SPSS (Model 7, 5,000 bootstrap samples) to examine whether PSI mediated the interaction between cuteness type and brand impression on brand evaluation. First, regarding the regression paths (see Table 2), the results indicated that the interaction between cuteness type and brand impression significantly predicted PSI (*B* = 0.47, SE = 0.17, *p* < 0.001). Additionally, PSI significantly predicted brand evaluation (*B* = 0.67, SE = 0.05, *p* < 0.001), controlling for the independent variable. These significant paths provided the preliminary basis for the moderated mediation effect.

**Table 2:** Regression analysis of mediating effects.

Predictor	Mediator = PSI		DV = Brand Evaluation	
	*B*	SE_B_	*B*	SE_B_
Cuteness type	−0.56***	0.12	0.06	0.08
Brand impression	−0.55***	0.12	-	-
Cuteness type x Brand impression	0.47***	0.17	-	-
PSI	-		0.67***	0.05

*N* = 321. Cuteness type was coded as 0 (kindchenschema) and 1 (whimsical). Brand impression was coded as 0 (warmth) and 1 (competence). *p < 0.05; **p < 0.01; ***p < 0.001.

As detailed in Table 3, the analysis revealed a significant index of moderated mediation [Index = 0.68, 95% CI (0.46, 0.91)]. The conditional indirect effect was negative and significant for the warmth brand [Effect = −0.38, 95%CI (−0.56, −0.21)], suggesting that kindchenschema cuteness enhances brand evaluation through increased PSI. For the competence brand, the indirect effect was positive and significant [Effect = 0.30, 95% CI (0.16, 0.44)], suggesting that whimsical cuteness works better through PSI in this context. Thus, H3 was supported.

**Table 3:** Moderated mediation analysis.

	Path: cuteness type-PSI -brand evaluation					
Moderator	Conditional indirect effects			Index of moderated mediation		
	Coefficient	SE	95% CI	Index	SE	95% CI
Warmth	−0.38	0.09	−0.56, −0.21	0.68	0.12	0.46, 0.91
Competence	0.30	0.07	0.16, 0.44			

*N* = 321. Cuteness type was coded as 0 (kindchenschema) and 1 (whimsical). Brand impression was coded as 0 (warmth) and 1 (competence). *p < 0.05; **p < 0.01; ***p < 0.001.

## 5 Study 3: Generalizing the effect and testing the boundary condition of social exclusion

Study 3 had two primary goals: first, to test the generalizability of the matching effect observed in Study 2 using new product categories; and second, to examine social exclusion as a key boundary condition (H4). We employed a 2 (social exclusion: exclusion vs. inclusion) × 2 (brand impression: warmth vs. competence) × 2 (cuteness type: kindchenschema vs. whimsical) between-subjects factorial design.

### 5.1 Design and participants

A total of 503 participants (*M*_age_ = 28.74, *SD* = 8.06; 206 male) were recruited from a reputable online panel (Jianshu.cn). Participants who failed embedded attention checks were excluded prior to the analysis. All participants were randomly assigned to one of the eight experimental conditions.

### 5.2 Procedure and manipulations

The procedure began with the social exclusion manipulation. Participants were randomly assigned to either the exclusion or inclusion condition and completed a writing task based on the classic paradigm by Williams et al. (2000). Those in the exclusion condition were asked to recall and write about a personal experience of being rejected or excluded. Those in the inclusion condition wrote about an experience of being accepted or included. Afterward, they completed scales to check the manipulation and to measure their current mood state.

Next, participants were presented with brand stimulation. In order to enhance the universality of our findings, we use new product categories for brand impression manipulation. Warm brand is a fictional towel company (“Rou Rong”), representing low-involvement products. The competence brand was a fictional electric vehicle company (“Zhi Chuang”), representing a high-involvement, high-risk product. This selection enables us to test whether the matching effect extends at different levels of consumer participation. The operation of cuteness type is the same as that in previous studies. The remainder of the procedure mirrored Study 2. After viewing their assigned page for at least 30 s, participants completed a questionnaire with the same measures for dependent variables, manipulation checks, and demographics.

### 5.3 Variable measurement

The Social Exclusion Manipulation Check Scale draws on the research of Williams et al. (2000) and uses three items to measure feelings of exclusion (*α* = 0.91). Warmth perception (*α* = 0.88), competence perception (*α* = 0.86), kindchenschema cuteness (*α* = 0.81), whimsical cuteness (*α* = 0.80), as well as PSI (*α* = 0.81), brand evaluation (*α* = 0.72) were measured and studied identically to Study 2. In addition, mood state (*α* = 0.91) and brand familiarity were also measured as control variables, using the same methods as in Study 2. Demographic information was also collected.

### 5.4 Results

#### 5.4.1 Discriminant validity of the measurement scales

The three-factor model demonstrated excellent fit (χ^2^/df = 1.12, CFI = 0.99, RMSEA = 0.04) and was significantly superior to the two-factor model [Δχ^2^(2) = 153.71, *p* < 0.001] and one-factor model [Δχ^2^(3) = 170.99, *p* < 0.001]. The square root of the AVE for each construct (ranging from 0.71 to 0.85) exceeded its correlations with the other constructs, supporting discriminant validity.

#### 5.4.2 Manipulation checks

##### 5.4.2.1 Cuteness type

Participants in the kindchenschema condition reported higher kindchenschema perceptions (*M* = 4.47, *SD* = 0.83) than those in the whimsical condition (*M* = 3.99, *SD* = 0.89), *t* (501) = 6.57, *p* < 0.001. Conversely, the whimsical group rated their stimuli as more whimsically cute (*M* = 4.41, *SD* = 0.85) than the kindchenschema group did (*M* = 3.44, *SD* = 0.78), *t* (501) = −10.40, *p* < 0.001.

##### 5.4.2.2 Brand impression

The warmth brand was perceived as significantly warmer (*M* = 5.85, *SD* = 0.75) than the competence brand (*M* = 4.67, *SD* = 1.21), *t*(429.27) = 13.27, *p* < 0.001. The competence brand was also rated as more competent (*M* = 5.27, *SD* = 1.02) than the warmth brand (*M* = 4.68, *SD* = 0.95), *t* (501) = −6.79, *p* < 0.001.

##### 5.4.2.3 Social exclusion

Participants in the exclusion condition reported significantly higher feelings of social exclusion (*M* = 5.06, *SD* = 0.98) than those in the inclusion condition (*M* = 1.96, *SD* = 0.55), *t* (395.66) = −43.75, *p* < 0 0.001.

#### 5.4.3 Replication of the matching effect

To test the robustness of our findings, we reran our analyses from Study 2, omitting the social exclusion variable. First, a 2×2 ANOVA replicated the pivotal “cuteness type × brand impression” interaction on brand evaluation, *F*(1, 499) = 19.83, *p* < 0.001, *η^2^* = 0.04. Simple effects analysis further confirmed that kindchenschema cuteness was preferred for warmth brands (*M* = 5.37 vs. 5.22), *F*(1, 499) = 18.25, *p* < 0.001, whereas whimsical cuteness was preferred for competence brands (*M* = 5.32 vs. 5.16), *F*(1, 499) = 11.22, *p* = 0.001. Moreover, after accounting for perceived cuteness, the “Cuteness Type × Brand Impression” interaction effect remained significant, *F*(1, 499) = 25.10, *p* < 0.001. Second, the moderated mediation model (H3) also proved robust. The overall index remained significant [Index = 0.11, SE = 0.03, 95% CI (0.06, 0.17)]. Consistent with our primary findings, the conditional indirect effect of cuteness type on brand evaluation (via PSI) was negative for warmth brands [Effect = −0.06, 95% CI (−0.09, −0.03)] and positive for competence brands [Effect = 0.05, 95% CI (0.02, 0.09)], supporting H3.

#### 5.4.4 The moderating role of social exclusion

A 2 (social exclusion) × 2 (cuteness type) × 2 (brand impression) ANOVA was conducted to test H4 and RQ. The analysis yielded a significant three-way interaction, *F* (1, 495) = 2.59, *p* = 0.036, *η^2^* = 0.02, indicating that social exclusion moderated the matching effect between cuteness type and brand impression.

**Figure 3:**
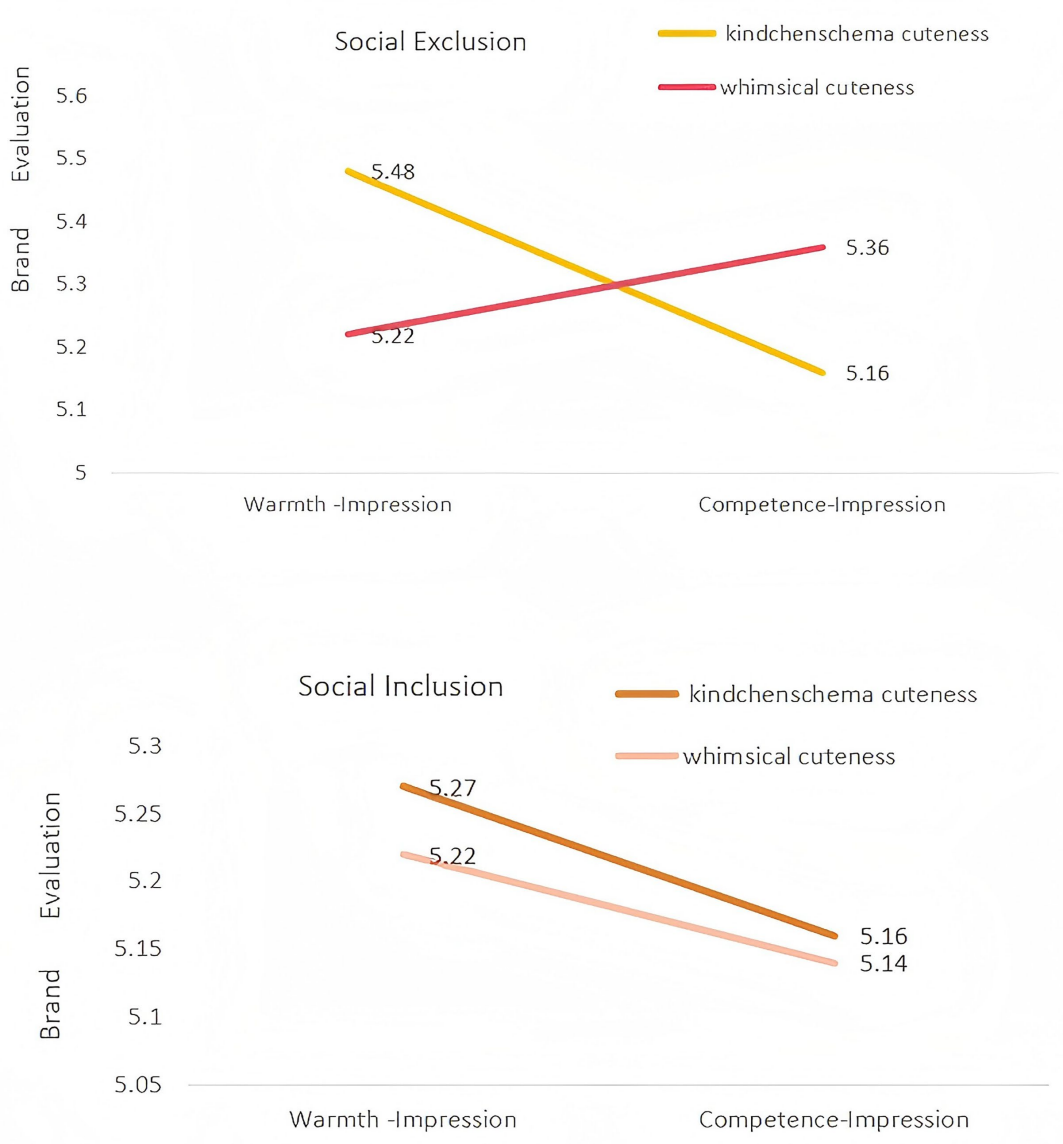
Three-way effects among cuteness type, brand impression, and social exclusion on brand evaluation. *N*=503. Cuteness type was coded as 0 (kindchenschema) and 1 (whimsical). Brand impression was coded as 0 (warmth) and 1 (competence). Social exclusion was coded as 0 (exclusion) and 1 (inclusion). *p<0.05; **p< 0.01; ***p<0.001.

To probe this interaction, we first examined the social exclusion condition. As predicted by H4, the “cuteness type × brand impression” interaction was highly significant, *F* (1, 248) = 20.78, *p* < 0.001, *η^2^* = 0.08. Simple effects analysis revealed that excluded participants evaluated warmth brands more favorably when paired with kindchenschema cuteness (*M* = 5.48) than with whimsical cuteness (*M* = 5.22), *p* < 0.001. Conversely, they rated competence brands higher when matched with whimsical cuteness (*M* = 5.36) versus kindchenschema cuteness (*M* = 5.16), *p* = 0.004. These results support H4, suggesting that excluded consumers seek belonging via a warmth-kindchenschema match and may seek emotional repair via a competence-whimsical match. In contrast, and in answer to RQ, the two-way interaction was not significant for the social inclusion group, *F* (1, 247) = 3.08, *p* = 0.081. This suggests the matching effect is not activated when the need for belonging is met (Figure 3).

## 6 Conclusion

### 6.1 Discussion

Through three experiments, this study systematically investigates the psychological mechanisms by which different types of cuteness influence brand evaluation on social media, and examines key boundary conditions of these effects. Our findings provide a comprehensive perspective for the complexity of cute marketing, and reveal that its effectiveness does not depend on cuteness itself, but on the fit between cuteness type, brands and consumers’ psychological needs in a given context.

First, we find that the effectiveness of a cute appeal is highly contingent on its alignment with the brand impression. Study 1 established a key baseline: in the context without a brand, there was no significant difference in the ability of kindchenschema and whimsical cuteness to evoke PSI. However, their effects diverged significantly when situated within different brand contexts. Study 2 and 3 consistently showed that brands perceived as “warm” benefit more from kindchenschema cuteness, whereas brands perceived as “competent” are better suited to whimsical cuteness. This finding helps to explain the inconsistent results of cute marketing activities in practice and extends previous studies on the matching effect between cuteness and product categories (Ye and Shi, 2020; Feng et al., 2022; Ye et al., 2023).

Second, our study reveals that a successful match enhances consumers’ PSI with the brand. We propose that a cute appeal consistent with the personified image of the brand is more likely to form an emotional connection with consumers. The improved PSI level will correspondingly bring more favorable overall brand evaluation. This results confirms the intermediary role of PSI in digital marketing (Shen, 2020; Golonka et al., 2023).

Finally, this study defines an important boundary condition of the matching effect: social exclusion as a moderator will significantly amplify the effect, but the effect is not significant in the non excluded individuals. This shows that the threatened state of the need for belonging will increase individuals’ sensitivity to social cues and make them actively seek compensation. Accordingly, the matching of “warmth-kindchenschema” can play a particularly prominent social compensation function by transmitting care and intimacy. This finding not only supports the Compensatory Consumption Theory (Sivanathan and Pettit, 2010), but also echoes the previous literature on the preference of excluded individuals for warm brands (Chen and Yang, 2017; Chou et al., 2022; Su and Li, 2023). It is worth noting that we also observed that excluded individuals have similar preferences for the “competence-whimsical” combination. This shows that for these consumers, in addition to directly making up for the lack of a sense of belonging, humor and novel elements can also be used to regulate emotions, which may also be an alternative regulation mechanism.

It should be noted that although the core matching effect and its mediating path have been confirmed to be significant in many experiments, the observed effect size was in the small to moderate range. This means that in the social media information stream, the psychological impact of one-time and properly matched cute content exposure is subtle rather than transformative. This points out the limit of cute marketing effect: it is a variable highly dependent on the situation, and its value can be fully displayed only under the long-term brand content ecology and specific user psychological background.

### 6.2 Theoretical implications

This study aims to solve the practical paradox of “when cute marketing works”. By integrating the SCM, consistency theory, and PSI theory, and introducing social exclusion as a boundary condition, we constructed a comprehensive model that explains the mechanism of cuteness becoming effective in the relational context of social media. It provides new insights on the following interrelated levels.

First, through an analysis of cuteness as a distinct relational signal, this study proposes a conceptual link to the “Cuteness-Impression Consistency Model.”

Although the matching effect in the literature (e.g., Feng et al., 2022) is well documented, we argue that cuteness is not merely an aesthetic attribute, but a powerful relationship invitation signal. Kindchenschema cuteness and whimsical cuteness are rooted in evolutionarily formed caregiving instincts and cognitively driven humor perception, respectively. Their core function is to activate specific relationship scripts. Therefore, matching depends on the consistency between the brand’s relationship invitation and its projected social role. This perspective extends the application of consistency theory from superficial feature similarity (Aggarwal and McGill, 2007) to the deeper level of social cognition and relationship construction. It explains why not all forms of cuteness are effective, and advances the application of consistency theory in digital marketing from simply determining “whether there is a match” to a more predictive stage of identifying “what kind of functional match is needed”.

Second, it elucidates the complete psychological path from cognitive congruence to relationship building, establishing PSI’s mediating role as a conditional outcome. Although existing studies generally regard PSI as the automatic result of social interaction (Labrecque, 2014; Shen, 2020), this study explicitly assumes and verifies PSI as the results of conditional relationship depending on matching quality. The psychological pathway can be interpreted as follows: when the cuteness type and brand impression achieve “relational signal-social role” congruence, it first optimizes the consumer’s processing fluency. The pleasure derived from this fluency is unconsciously attributed to the attractiveness and authenticity of the personified brand. This enhanced perception of charm and authenticity lowers the consumer’s psychological defenses, making them more willing to accept the brand’s invitation for relational interaction, thereby leading to emotional investment and the development of one-sided intimacy.

Third, this study examines social exclusion as a moderating factor, which helps clarify the psychological conditions under which the matching effect works. The results show that the effect is significantly stronger mainly among individuals who feel socially excluded. This is more than just a simple interaction; it offers an explanatory account of the underlying motivation within the theoretical model. Specifically, the reason why consistency between cuteness types and brand impressions promotes PSI and positive evaluations is that it can address certain psychological needs—particularly the need for belonging among those who lack it. These findings extend compensatory consumption theory by incorporating a social-motivational boundary condition. For the warm-kindchenschema match, it directly provides emotional solace and social substitution; for the competent–whimsical match, it offers emotion regulation through cognitive pleasure.

Fourth, this study applies the SCM to anthropomorphic brand communication, offering a better way to manage brand personality. We turn the SCM dimensions of warmth and competence into relationship roles to guide the use of cuteness in marketing. The research shows the link between the two types of cuteness and these SCM dimensions. This gives a theoretical path for brands—especially competent brands facing the “cuteness paradox” (Shin and Mattila, 2021).

### 6.3 Managerial implications

This study suggests that managers should focus less on whether to use cuteness, and more on how to use the right type. Our findings show that matching the cuteness type with the brand’s impression is a practical and valuable part of content design. Although the impact of a single post might be small in a busy feed, this consistency becomes important when maintained over time as part of a broader marketing strategy.

First, for brands seen as warm (e.g., in food or hospitality), kindchenschema cuteness—using soft visuals and affectionate language—works best. For brands viewed as competent (e.g., in technology or finance), whimsical cuteness—using humor and wit—is generally the better fit. A mismatch risks making the brand feel inauthentic and confusing the message.

Second, managers should look beyond simple numbers like page views and evaluate success based on relationship building. PSI is a useful indicator for this. The goal is to shift from corporate broadcasting to genuine interaction, helping consumers feel they are connecting with a relatable personality rather than an impersonal organization.

Third, cute marketing can help with targeting specific groups. We found that consumers who feel socially excluded respond more positively to matching cute appeals. Marketers can identify these users through online behavioral cues (such as their language or community engagement) and adjust content accordingly. For this group, a well-matched cute strategy acts as “emotional comfort,” building a type of loyalty that goes beyond simple transactions. This makes cuteness a practical tool for brands aiming to build community and emotional connections.

### 6.4 Limitations and future research

First, we use laboratory experiments to ensure internal validity while limiting the external validity we find. Forced contact with static images is an idealization of the dynamic social media environment. In the future, we should test our model in more real environments. For example, the A/B test is used to measure how the matching of cuteness type and brand impression on a real brand account affects the real-world consumer behavior, such as sharing and conversion. Meanwhile, methods like eye-tracking could offer direct evidence of attention and cognitive fluency during browsing, moving beyond self-report measures.

Second, as for the manipulation of cuteness type, although we strictly abide by the established paradigm, we must admit that the whimsical cuteness and kindchenschema cuteness may not be completely orthogonal visual design. In the future, neural marketing methods (e.g., fMRI or EEG) can be used to more accurately verify the uniqueness of these structures by distinguishing the neural activation patterns related to “emotional cultivation response” and “cognitive humor response”.

Third, as noted in the method section, we use different product categories to manipulate brand impressions. This approach, while grounded in impression formation theory and prior research, inherently couples product category with the intended psychological construct. Although the consistent results of different product categories in our study support the robustness of brand impression effect, future research should aim to replicate these findings while keeping the product categories constant to more strictly isolate the influence of brand variables.

Furthermore, although our theoretical framework assumes that processing fluency and perceived authenticity are the cognitive antecedents of PSI, the current research does not empirically measure these mediating variables. In the future, a series of mediation models can be introduced to directly measure processing fluency, perceived authenticity or anthropomorphism, so as to provide an empirical test of the detailed psychological path from impression consistency to PSI.

Moreover, our study focused on positive outcomes. A crucial next step is to investigate the potential “dark side” of cuteness. For example, during service failures, cute appeal may be seen as trivial and inappropriate, causing damage to the brand, especially for a competence-centered brand.

Finally, our study was conducted in a single cultural context. The East Asian concept of “moral cuteness,” where prosocial corporate actions are viewed as “cute,” offers a fascinating direction for cross-cultural research.

## Data Availability

The original contributions presented in the study are included in the article/Supplementary material, further inquiries can be directed to the corresponding author/s.

## Footnotes

1 Available online at: www.wjx.cn.

## References

[ref1] Aaker J. L. (1997). Dimensions of brand personality. J. Mark. Res. 34, 347–356. doi: 10.1177/002224379703400304

[ref3] Aggarwal P. McGill A. L. (2007). Is that car smiling at me? Schema congruity as a basis for evaluating anthropomorphized products. J. Consum. Res. 34, 468–479. doi: 10.1086/518544

[ref4] Baumeister R. F. Leary M. R. (1995). The need to belong: desire for interpersonal attachments as a fundamental human motivation. Psychol. Bull. 117, 497–529. doi: 10.1037/0033-2909.117.3.497, 7777651

[ref5] Bellezza S. Gino F. Keinan A. (2014). The red sneakers effect: inferring status and competence from signals of nonconformity. J. Consum. Res. 41, 35–54. doi: 10.1086/674870

[ref6] Beverland M. B. Farrelly F. J. (2010). The quest for authenticity in consumption: consumers’ purposive choice of authentic cues to shape experienced outcomes. J. Consum. Res. 36, 838–856. doi: 10.1086/615047

[ref7] Bruckdorfer R. E. Büttner O. B. (2025). Cute package, sweet taste? The effects of Kindchenschema food packaging on consumers. Int. J. Res. Mark. doi: 10.1016/j.ijresmar.2025.11.001

[ref8] Chandon P. Wansink B. Laurent G. (2000). A benefit congruency framework of sales promotion effectiveness. J. Mark. 64, 65–81. doi: 10.1509/jmkg.64.4.65.18071

[ref9] Chao Y. L. (2022). An eco-label can matter more than buying green: an experiment on consumers' recycling behaviour after tasting eco-labeled coffee. Int. J. Sustain. Dev. Plan. 17, 1355–1365. doi: 10.18280/ijsdp.170433

[ref10] Chen C.-H. Jia X.-Y. (2023). Research on the influence of the baby schema effect on the cuteness and trustworthiness of social robot faces. Int. J. Adv. Robot. Syst. 20. doi: 10.1177/17298806231168486

[ref11] Chen Z. Yang G. (2017). Which kind of brand anthropomorphic image is more preferred: the moderating effect and boundary of the need to belong. Nankai Bus. Rev. 20, 135–143. doi: 10.3969/j.issn.1008-3453.2017.03.013

[ref13] Chou H. Y. Chu X. Y. Chen T. C. (2022). The healing effect of cute elements. J. Consum. Aff. 56, 565–596. doi: 10.1111/joca.12414

[ref14] Dhar R. Wertenbroch K. (2000). Consumer choice between hedonic and utilitarian goods. J. Mark. Res. 37, 60–71. doi: 10.1509/jmkr.37.1.60.18718

[ref15] Dibble J. L. Hartmann T. Rosaen S. F. (2016). Parasocial interaction and parasocial relationship: conceptual clarification and a critical assessment of measures. Hum. Commun. Res. 42, 21–44. doi: 10.1111/hcre.12063

[ref16] Epley N. Waytz A. Cacioppo J. T. (2007). On seeing human: a three-factor theory of anthropomorphism. Psychol. Rev. 114, 864–886. doi: 10.1037/0033-295X.114.4.864, 17907867

[ref17] Escalas J. E. Bettman J. R. (2017). Connecting with celebrities: how consumers appropriate celebrity meanings for a sense of belonging. J. Advert. 46, 297–308. doi: 10.1080/00913367.2016.1274925

[ref18] Feng W. Xu Y. Huang H. Wang T. (2022). Sweetly cute or whimsically cute? The effect of luxury brands’ cute style on consumer preference. Acta Psychol. Sin. 54, 313–330. doi: 10.3724/SP.J.1041.2022.00313

[ref19] Fiske S. T. Neuberg S. L. (1990). A continuum of impression formation, from category-based to individuating processes: influences of information and motivation on attention and interpretation. Adv. Exp. Soc. Psychol. 23, 1–74. doi: 10.1016/S0065-2601(08)60317-2

[ref20] Gardner W. L. Pickett C. L. Brewer M. B. (2000). Social exclusion and selective memory: how the need to belong influences memory for social events. Personal. Soc. Psychol. Bull. 26, 486–496. doi: 10.1177/0146167200266007

[ref21] Gidaković P. Szőcs I. Diamantopoulos A. Florack A. Egger M. Žabkar V. (2021). The interplay of brand, brand origin and brand user stereotypes in forming value perceptions. Br. J. Manag. 33, 1924–1944. doi: 10.1111/1467-8551.12552

[ref22] Golonka E. M. Jones K. M. Sheehan P. Pandža N. B. Paletz S. B. F. Rytting C. A. . (2023). The construct of cuteness: a validity study for measuring content and evoked emotions on social media. Front. Psychol. 14:1068373. doi: 10.3389/fpsyg.2023.1068373, 36935945 PMC10020712

[ref23] Gorn G. J. Jiang Y. Johar G. V. (2008). Babyfaces, trait inferences, and company evaluations in a public relations crisis. J. Consum. Res. 35, 36–49. doi: 10.1086/529533

[ref24] Gretry A. Horváth C. Belei N. Van Riel A. C. R. (2017). "Don't pretend to be my friend!" when an informal brand communication style backfires on social media. J. Bus. Res. 74, 77–89. doi: 10.1016/j.jbusres.2017.01.012

[ref25] Halberstadt J. B. (2006). The generality and ultimate origins of the attractiveness of prototypes. Personal. Soc. Psychol. Rev. 10, 166–183. doi: 10.1207/s15327957pspr1002_5, 16768653

[ref26] Horton D. Wohl R. R. (1956). Mass communication and Para-social interaction. Psychiatry 19, 215–229. doi: 10.1080/00332747.1956.11023049, 13359569

[ref27] Jeong H. J. Kim J. (2021). Human-like versus me-like brands in corporate social responsibility: the effectiveness of brand anthropomorphism on social perceptions and buying pleasure of brands. J. Brand Manag. 28, 32–47. doi: 10.1057/s41262-020-00212-8

[ref28] Jörling M. Böhm R. Paluch S. (2020). Mechanisms and consequences of anthropomorphizing autonomous products. Schmalenbach. Bus. Rev 72, 485–510. doi: 10.1007/s41464-020-00100-3

[ref30] Kervyn N. Fiske S. T. Malone C. (2012). Brands as intentional agents framework: how perceived intentions and ability can map brand perception. J. Consum. Psychol. 22, 166–176. doi: 10.1016/j.jcps.2011.09.006, 24403815 PMC3882007

[ref31] Kringelbach M. L. Stark E. A. Alexander C. Bornstein M. H. Stein A. (2016). On cuteness: unlocking the parental brain and beyond. Trends Cogn. Sci. 20, 545–558. doi: 10.1016/j.tics.2016.05.003, 27211583 PMC4956347

[ref32] Labrecque L. I. (2014). Fostering consumer–brand relationships in social media environments: the role of parasocial interaction. J. Interact. Mark. 28, 134–148. doi: 10.1016/j.intmar.2013.12.003

[ref33] Lazarus R. S. (1991). Progress on a cognitive-motivational-relational theory of emotion. Am. Psychol. 46, 819–834. doi: 10.1037/0003-066X.46.8.819, 1928936

[ref35] Li Y. Eastman J. (2024). Does cuteness enhance luxury brand equity? Exploring the effect of perceived uniqueness. Psychol. Mark. 41, 2298–2309. doi: 10.1002/mar.22053

[ref36] Li B. Nan Y. Yao R. (2022). Warmth or competence? The effects of cool and cuteness on the perceived quality of digital products. Asia Pac. J. Mark. Logist. 34, 1880–1904. doi: 10.1108/APJML-06-2021-0413

[ref37] Li F. Zhou Z. (2023). The interaction effect of endorser type and destination stereotype on destination evaluation. J. Travel Tour. Mark. 40, 878–893. doi: 10.1080/10548408.2023.2296661

[ref38] Lorenz K. (1943). Die angeborenen Formen möglicher Erfahrung. Z. Tierpsychol. 5, 235–409. doi: 10.1111/j.1439-0310.1943.tb00655.x

[ref39] Lv X. Liu Y. Luo J. Liu Y. Li C. (2021). Does a cute artificial intelligence assistant soften the blow? The impact of cuteness on customer tolerance of assistant service failure. Ann. Tour. Res. 87:103114. doi: 10.1016/j.annals.2020.103114

[ref40] Maner J. K. DeWall C. N. Baumeister R. F. Schaller M. (2007). Does social exclusion motivate interpersonal reconnection? Resolving the "porcupine problem.". J. Pers. Soc. Psychol. 92, 42–55. doi: 10.1037/0022-3514.92.1.42, 17201541

[ref41] Messer U. Pape D. Lukas N. Peters L. (2024). From cute to incompetent: the impact of anthropomorphic design on responsibility attribution in autonomous driving. Proceedings of the 57th Hawaii International Conference on System Sciences. Available online at: https://aisel.aisnet.org/hicss-57/da/smart_mobility/2

[ref42] Nass C. Moon Y. (2000). Machines and mindlessness: social responses to computers. J. Soc. Issues 56, 81–103. doi: 10.1111/0022-4537.00153

[ref43] Nenkov G. Y. Scott M. L. (2014). "so cute I could eat it up": priming effects of cute products on indulgent consumption. J. Consum. Res. 41, 326–341. doi: 10.1086/676581

[ref44] Nittono H. Ihara N. (2017). Psychophysiological responses to kawaii pictures with or without baby schema. SAGE Open 7. doi: 10.1177/2158244017709321

[ref45] Osgood C. E. Tannenbaum P. H. (1955). The principle of congruity in the prediction of attitude change. Psychol. Rev. 62, 42–55. doi: 10.1037/h0048153, 14357526

[ref46] Park C. W. Jaworski B. J. MacInnis D. J. (1986). Strategic brand concept-image management. J. Mark. 50, 135–145. doi: 10.1177/002224298605000401

[ref47] Perse E. M. Rubin R. B. (1989). Attribution in social and parasocial relationships. Commun. Res. 16, 59–77. doi: 10.1177/009365089016001003

[ref48] Puzakova M. Aggarwal P. (2018). Brands as rivals: consumer pursuit of distinctiveness and the role of brand anthropomorphism. J. Consum. Res. 45, 869–888. doi: 10.1093/jcr/ucy035

[ref50] Reber R. Schwarz N. Winkielman P. (2004). Processing fluency and aesthetic pleasure: is beauty in the perceiver’s processing experience? Personal. Soc. Psychol. Rev. 8, 364–382. doi: 10.1207/s15327957pspr0804_3, 15582859

[ref51] Rubin A. M. Perse E. M. Powell R. A. (1985). Loneliness, parasocial interaction, and local television news viewing. Hum. Commun. Res. 12, 155–180. doi: 10.1111/j.1468-2958.1985.tb00071.x

[ref52] Schoch S. Nikitin J. Freund A. M. (2015). Why do(n't) you like me? The role of social approach and avoidance motives in attributions following social acceptance and rejection. Motiv. Emot. 39, 680–692. doi: 10.1007/s11031-015-9482-1

[ref53] Septianto F. Kwon J. (2022). Too cute to be bad? Cute brand logo reduces consumer punishment following brand transgressions. Int. J. Res. Mark. 39, 1108–1126. doi: 10.1016/j.ijresmar.2021.12.006

[ref54] Shen B. (2020). Creating a parasocial relationship on social media: luxury brands playing cute in China. Asian J. Commun. 30, 494–514. doi: 10.1080/01292986.2020.1840601

[ref55] Sherman G. D. Haidt J. (2011). Cuteness and disgust: the humanizing and dehumanizing effects of emotion. Emot. Rev. 3, 245–251. doi: 10.1177/1754073911402396

[ref57] Shin J. Mattila A. S. (2021). Aww effect: engaging consumers in "non-cute" prosocial initiatives with cuteness. J. Bus. Res. 126, 209–220. doi: 10.1016/j.jbusres.2020.11.046

[ref58] Sivanathan N. Pettit N. C. (2010). Protecting the self through consumption: status goods as affirmational commodities. J. Exp. Soc. Psychol. 46, 564–570. doi: 10.1016/j.jesp.2010.01.006

[ref59] Stavropoulos K. K. M. Alba L. A. (2018). "it's so cute I could crush it!": understanding neural mechanisms of cute aggression. Front. Behav. Neurosci. 12:300. doi: 10.3389/fnbeh.2018.00300, 30564109 PMC6288201

[ref60] Su Q. Li F. S. (2023). How cute mascots affect relationships with tourism destinations: a moderated mediation model. Tour. Manag. 99:104782. doi: 10.1016/j.tourman.2023.104782

[ref61] Suci A. Wang H. C. (2023). Can whimsically cute packaging overcome young consumer product unfamiliarity? Mark. Intell. Plan. 41, 574–592. doi: 10.1108/MIP-05-2022-0201

[ref62] Sun X. Cheng Y. Zhang M. (2025). The influence of cuteness types and sound characteristics of in-vehicle robots on user trust and experience in automatic driving error reporting scenarios. Kybernetes. doi: 10.1108/K-06-2024-1576

[ref63] Wang T. Mukhopadhyay A. Patrick V. M. (2017). Getting consumers to recycle now! When and why cuteness appeals influence prosocial and sustainable behavior. J. Public Policy Mark. 36, 269–283. doi: 10.1509/jppm.16.089

[ref64] Warren C. McGraw A. P. (2016). When does humorous marketing hurt brands? J. Mark. Behav. 2, 39–67. doi: 10.1561/107.00000027

[ref9001] Watson D. Clark L. A. Tellegen A. (1988). Development and validation of brief measures of positive and negative affect: The PANAS scales. J. Pers. Soc. Psychol. 54, 1063–1070. doi: 10.1037//0022-3514.54.6.1063 3397865

[ref65] Waytz A. Cacioppo J. T. Epley N. (2010). Who sees human? The stability and importance of individual differences in anthropomorphism. Perspect. Psychol. Sci. 5, 219–232. doi: 10.1177/1745691610369336, 24839457 PMC4021380

[ref9002] Williams K. D. (2007). Ostracism. Annu. Rev. Psychol. 58, 425–452. doi: 10.1146/annurev.psych.58.110405.085641 16968209

[ref8001] Williams K. D. Cheung C. K. Choi W. (2000). Cyberostracism: Effects of being ignored over the Internet. J. Pers. Soc. Psychol. 79, 748–762. doi: 10.1037/0022-3514.79.5.748 11079239

[ref66] Winkielman P. Cacioppo J. T. (2001). Mind at ease puts a smile on the face: psychophysiological evidence that processing facilitation increases positive affect. J. Pers. Soc. Psychol. 81, 989–1000. doi: 10.1037/0022-3514.81.6.989, 11761320

[ref67] Winkielman P. Halberstadt J. Fazendeiro T. Catty S. (2006). Prototypes are attractive because they are easy on the mind. Psychol. Sci. 17, 799–806. doi: 10.1111/j.1467-9280.2006.01785.x, 16984298

[ref9003] Wu Y. Jiang J. (2019). Partner or servant: How anthropomorphized brand role releases the negative effect of social exclusion. J. Contemp. Mark. Sci. 2, 284–297. Available online at: http://www.jms.org.cn:8081/jms/EN/Y2018/V14/I1/44

[ref68] Xu X. Wang S. Liu J. Wang X. L. Morgan N. (2025). How does the cuteness of persuasive communication about sustainable tourism shape tourists’ environmentally responsible behavioral intentions? J. Sustain. Tour. doi: 10.1080/09669582.2025.2478187

[ref69] Yang K. (2023). How sajiao (playing cute) wins forgiveness: the effectiveness of emojis in rebuilding trust through apology. Discourse Commun. 17, 77–95. doi: 10.1177/17504813221123850

[ref70] Ye B. H. He J. Fong L. H. N. Li Z. Yan Y. Q. (2023). How does cuteness become the cue? Investigating the impact of cute destination spokespersons on tourist travel intention. J. Destin. Mark. Manag. 27:100758. doi: 10.1016/j.jdmm.2022.100758

[ref71] Ye W. Shi T. (2020). What kind of cute logo can enhance the perceived relative quality of a product? Nankai Bus. Rev. 23, 118–130. doi: 10.3969/j.issn.1008-3453.2020.01.012

[ref72] Yim A. Cui A. P. Walsh M. (2024). The role of cuteness on consumer attachment to artificial intelligence agents. J. Res. Interact. Mark. 18, 127–141. doi: 10.1108/JRIM-02-2023-0046

[ref73] Yoon N. Park W. Joo J. (2022). Dark side of cuteness: effect of whimsical cuteness on new product adoption. In: With design: reinventing design modes. (eds.) Bruyns G. Wei H., (Springer), 592–604. doi: 10.1007/978-981-19-4472-7_41

