# Supplementary material for: Cuteness conquest: how cuteness type and brand impression affect consumers’ brand evaluation in social media
Source: Front Psychol. 2026 Feb 27;17:1730776. doi: 10.3389/fpsyg.2026.1730776 (PMC12982413; doi:10.3389/fpsyg.2026.1730776)
Supplement: Supplementary file 1 [file Table_1.DOCX]

Appendix A Experimental Materials

**Study 1**


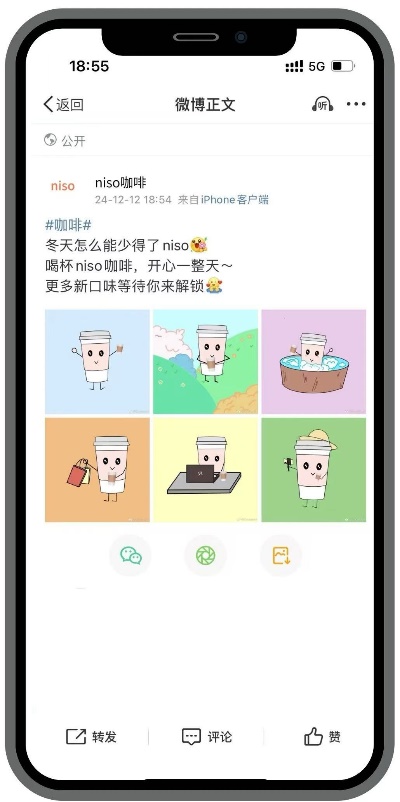

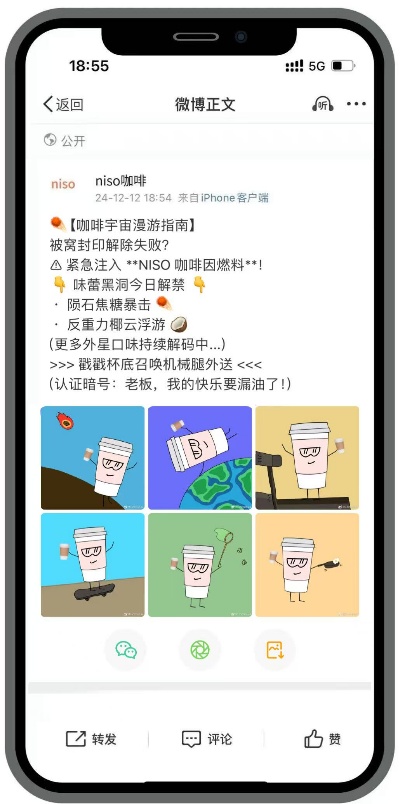

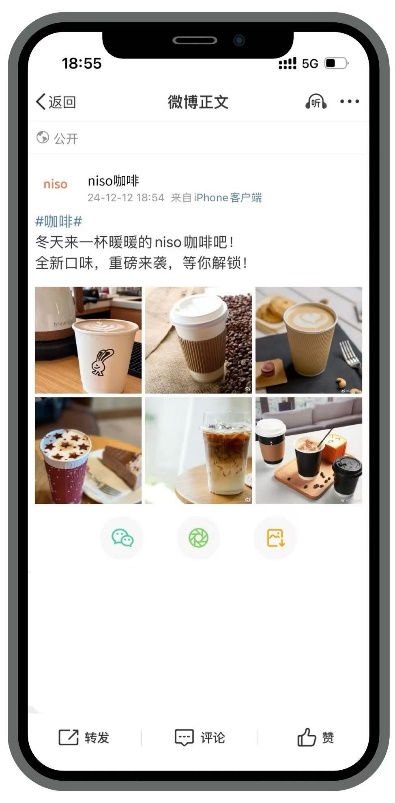


cute stimuli: kindchenschema, whimsical, vs. a non-cute contro

**Study 2**

Brand Introduction:

Brand Name: Mizhiyun

Product Positioning: Nourishers of Emotion and Life, Not Just Food Suppliers.

Slogan: Nature's Choice, Heavenly Sweetness

“Mizhiyun” is a honey brand that originates from nature and consistently adheres to the pursuit of quality and authenticity. The brand's mission is to bring consumers the purest and most natural honey, with every drop embodying the gifts of nature and the vitality of life. The honey sources of Melody of Nature come from high-quality floral sources around the world. In the production process, strict organic agricultural standards are followed, with no use of chemical fertilizers, pesticides, or antibiotics, ensuring the purity and safety of the honey. At the same time, advanced production processes and technologies are used to maximize the retention of natural enzymes, minerals, vitamins, and other nutritional components in the honey, allowing every bottle to exude the fragrance and sweetness of nature. Melody of Nature always adheres to a consumer-centered service philosophy and actively participates in public welfare and environmental protection activities, giving back to society through practical actions and spreading positive energy. Choosing Melody of Nature means choosing respect for nature and a love for life.


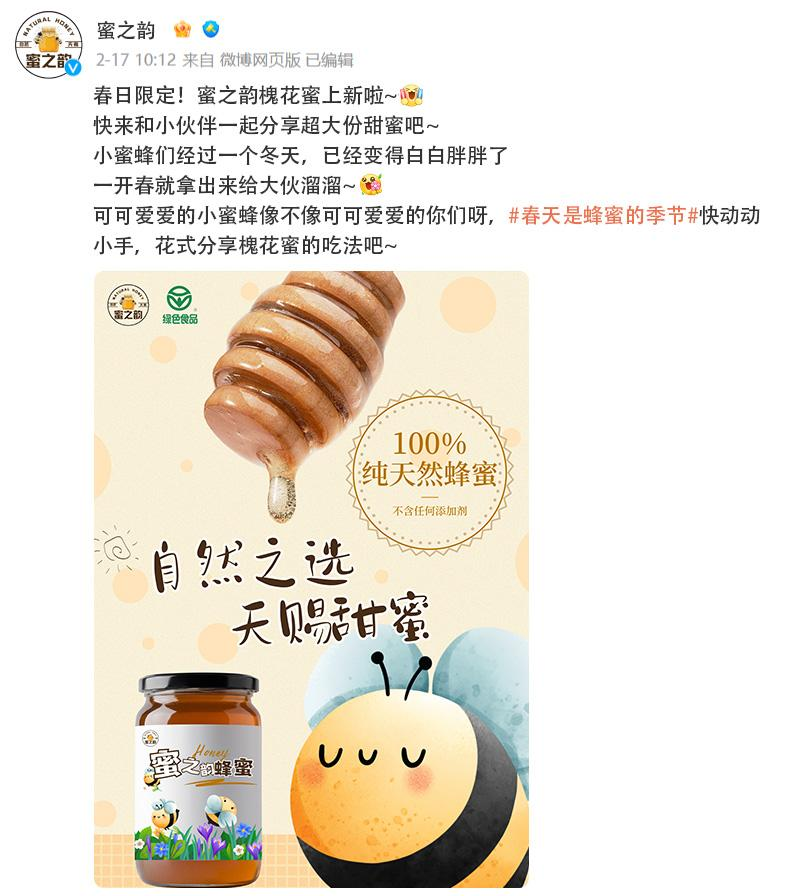

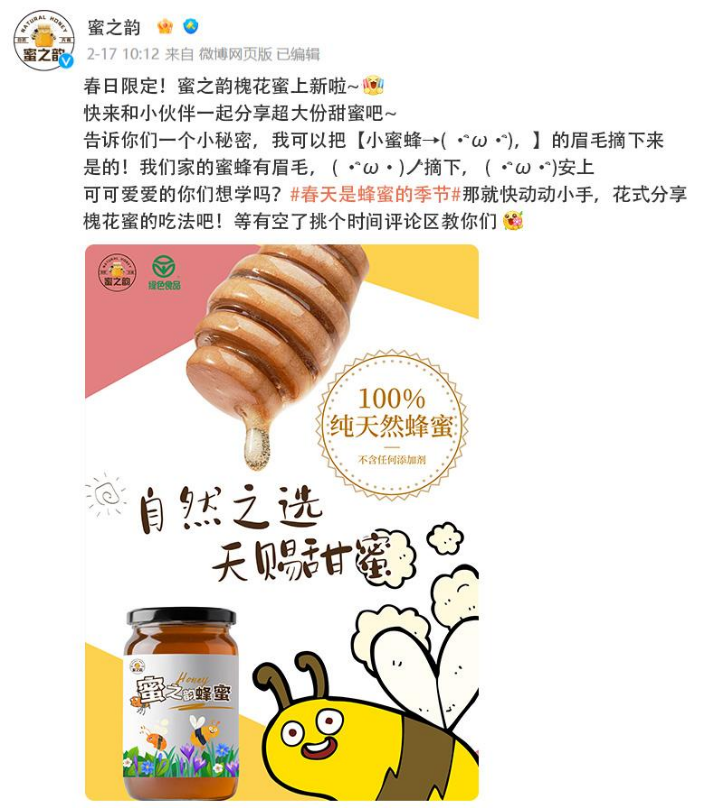
warmth* kindchenschema warmth* whimsical

Brand Introduction:

Brand Name: Zhihuijie

Product Positioning: An extension of the user's capabilities, the ultimate partner for professionals who pursue the pinnacle of efficiency.

Slogan: "Intelligence Converges, Future Within Reach"

"Zhihuijie" focuses on providing users with cutting-edge technology and superior performance smartphones. As a brand driven by innovation, it is committed to integrating the latest mobile communication technology, artificial intelligence, photographic art, and user experience design into every phone. The brand gathers top engineers and designers in the industry to ensure that its products lead in performance, speed, photographic capabilities, and battery life.

The products of "Intelligence Convergence" are equipped with the latest processors, offering seamless multitasking capabilities and an ultimate gaming experience. In terms of photography, they feature a multi-camera system and advanced image processing algorithms, capable of capturing delicate details and vivid colors in wide-angle shots, macro photography, and night mode. The body design uses high-quality materials and exquisite craftsmanship, while the phone screen employs the latest display technology to provide an immersive visual experience.

The brand believes that through continuous technological innovation and a deep understanding of user needs, "Intelligence Convergence" will lead the smartphone industry into a new era.


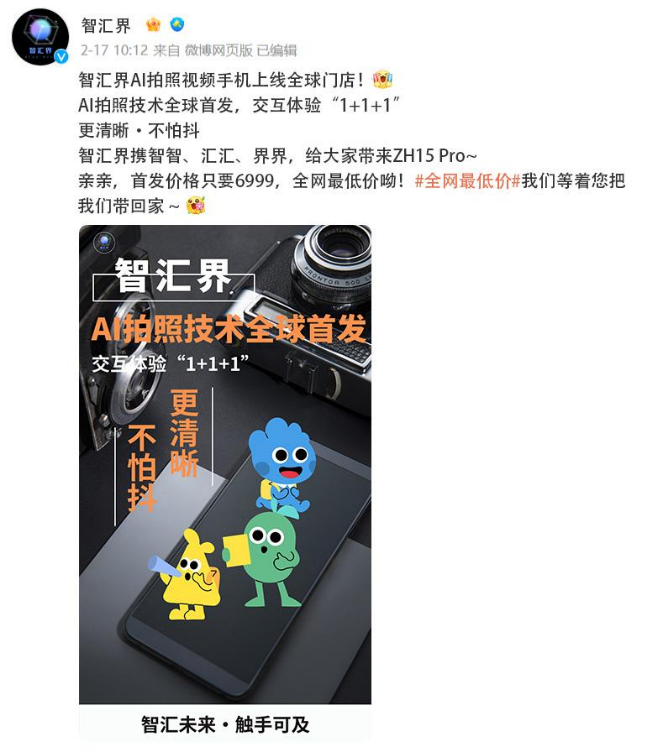

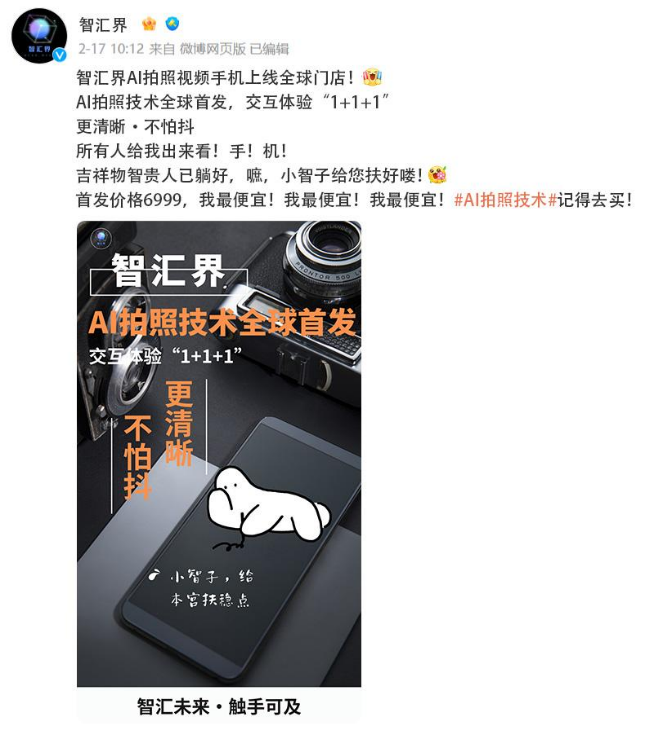


competence * kindchenschema competence * whimsical

**Study 3**

Brand Name: Mianrou

Product Positioning: Warmth and Healing Towel Brand

Slogan: Let Warmth Caress You

“Mianrou” is a towel brand dedicated to warmth and care, always committed to the ultimate pursuit of texture and comfort. The brand's mission is to bring the most considerate comfort to everyone who loves life, turning every wipe into a gentle hug that eases fatigue and returns you to softness.

Softness selects its cotton from the globally recognized golden cotton-growing regions, using only the finest long-staple cotton that has basked in over 2,000 hours of sunlight. In the weaving process, it adheres to twistless or low-twist yarn techniques, without any chemical additives that could harm the skin, ensuring every touch is safe and reassuring. Additionally, it employs a unique softening process that allows the cotton fibers to breathe fully, maximizing their natural fluffiness and softness, making each towel as light and skin-friendly as a cloud.

Softness believes that true warmth comes from attention to detail. We focus not only on product quality but also advocate a philosophy of slowing down and caring for oneself. We actively promote sustainable cotton farming to protect our shared home. Choosing Softness means choosing a way to treat yourself gently, filling every moment of life with warmth.


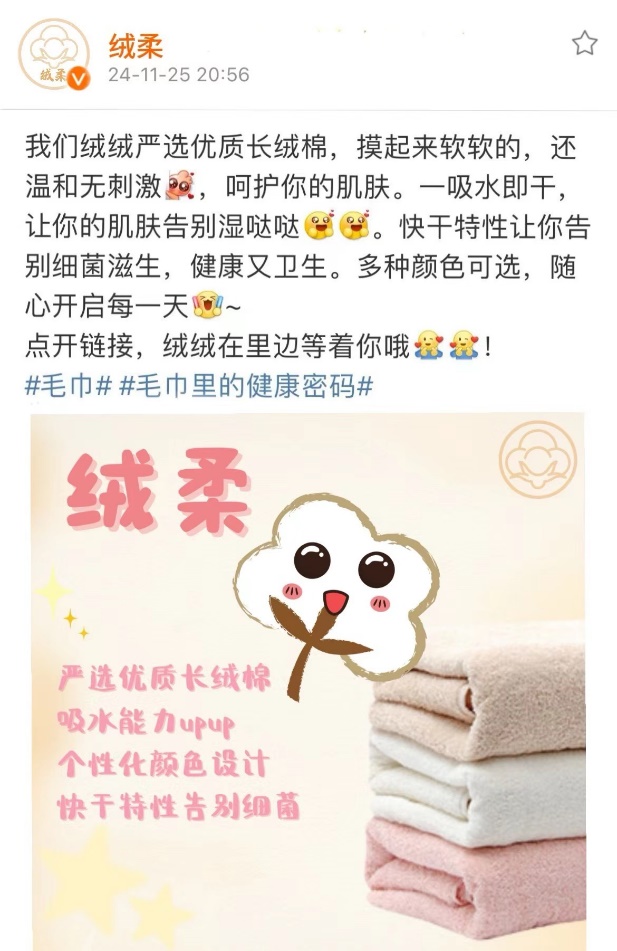

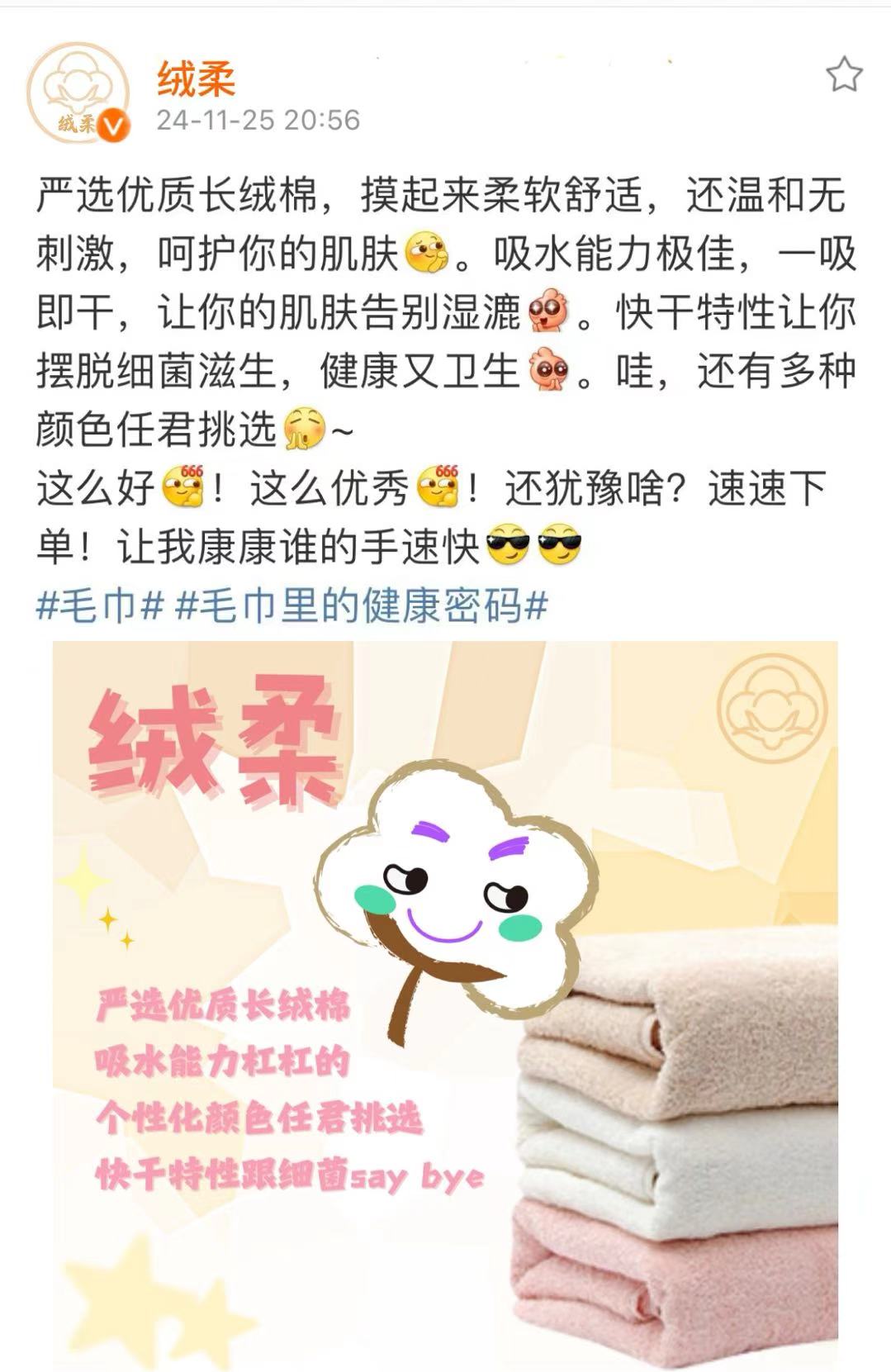


warmth* kindchenschema warmth* whimsical

Brand Name: ZhiChuang

Product Positioning: High-Performance Intelligent New Energy Vehicle Brand

Slogan: Intelligence Drives, Innovation Leads the Era

ZhiChuang is dedicated to creating new energy vehicles that integrate cutting-edge intelligent technology with ultimate driving performance. As a brand driven by technology, it is committed to incorporating the latest autonomous driving technology, energy management systems, human-machine interaction experiences, and power engineering into every vehicle. The brand has gathered top automotive engineers, algorithm experts, and aerodynamic designers from around the world to ensure that its products achieve industry-leading standards in power response, range, intelligent driving, and safety redundancy.

ZhiChuang's vehicles are equipped with self-developed high-power density electric drive systems and high-efficiency battery packs, providing zero-delay power response and astonishing acceleration performance. Whether navigating urban streets or racing on tracks, they deliver an unparalleled driving thrill. In terms of intelligent driving, the vehicles feature a multi-redundant sensor array (lidar, high-definition cameras, millimeter-wave radar) and a high-performance computing platform. Combined with advanced perception and decision-making algorithms, they can handle complex urban traffic conditions and high-speed cruising scenarios with ease, achieving high-level assisted driving. The body design follows aerodynamic principles, using one-piece stamping processes and lightweight high-strength materials. This not only creates a visually striking futuristic look but also ensures excellent energy efficiency and safety performance.


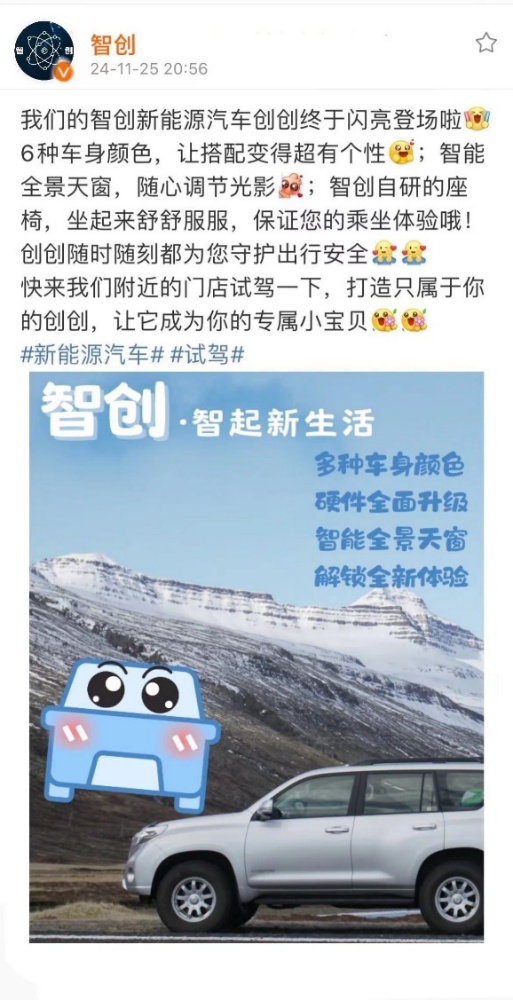

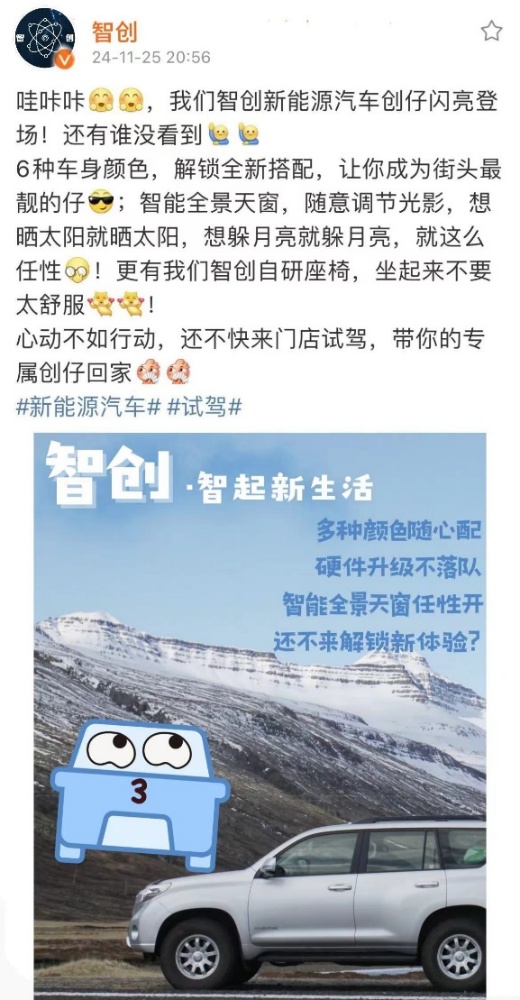


competence * kindchenschema competence * whimsical

Appendix B Measurement Items

Cuteness **(OC)^a^**

OC 1: After reading the brand page, I feel that the brand is cute

OC 2: After reading the brand page, I feel that the brand is adorable

OC 3: After reading the brand page, I feel that the brand is endearing

Kindchenschema Cuteness Perception（KCP）**^a^**

KCP1：After reading the brand page, I feel that the brand is vulnerable

KCP2 ：After reading the brand page, I feel that the brand is naive

KCP3：After reading the brand page, I feel that the brand is caretaking

Whimsical Cuteness Perception（WCP）**^a^**

WCP1：After reading the brand page, I feel that the brand is whimsical

WCP2 ：After reading the brand page, I feel that the brand is playful

WCP3：After reading the brand page, I feel that the brand is fun

Social Exclusion（SE）a

SE1：How much do you feel excluded?

SE2：How much do you feel rejected?

SE3：How much do you feel left out?

Moods **^a^**

M1: I am happy now

M2: I am in good mood now

M3: I am pleased now

M4: I am cheerful now

Brand Evaluation （BE）**^a^**

BE1. After reading the brand page, I really like that the brand.

BE2. After reading the brand page, I trust that the brand greatly.

BE3. After reading the brand page, I highly approve of the brand's quality.

BE4. After reading the brand page, I find that the brand very attractive.

BE5. After reading the brand page, I would buy products from this brand if possible.

Warmth Impression （WI）**^a^**

WI1: After reading the brand page, I feel that the brand is Warm

WI2: After reading the brand page, I feel that the brand is friendly

Competence Impression（CI）**^a^**

CI 1: After reading the brand page, I feel that the brand is competent

CI 2: After reading the brand page, I feel that the brand is capable

Parasocial Interaction（PI）**^a^**

PI1: I feel that the brand makes me comfortable, like a friend.

PI 2: I am willing to interact with the brand on Weibo.

PI 3: I believe that the brand understands my thoughts.

PI 4: I am willing to read Weibo content about that the brand.

Attitudes toward Brand Pages（ABP）**^a^**

ABP 1: To me the brand page on Weibo is interesting.

ABP 2: To me the brand page on Weibo is good.

ABP 3: To me the brand page on Weibo is clever.

ABP 4: To me the brand page on Weibo is useful.

ABP 5: T To me the brand page on Weibo is positive.

ABP 6: To me the brand page on Weibo is pleasant.

a 1= Strongly disagree 7= Strongly agree
